# Effect of Silicone Rubbers on the Properties of RDX-Based PBXs and Their Application in the Explosive Hardening of Steel

**DOI:** 10.3390/ma18102311

**Published:** 2025-05-15

**Authors:** Konrad Szydło, Agnieszka Stolarczyk, Tomasz Jarosz, Barbara Lisiecka, Sylwia Waśkiewicz, Krzysztof Lukaszkowicz, Klaudiusz Gołombek, Jakub Polis, Mateusz Polis

**Affiliations:** 1 Explosive Techniques Research Group, Łukasiewicz Research Network-Institute of Industrial Organic Chemistry, 42-693 Krupski Młyn, Poland; konrad.szydlo@ipo.lukasiewicz.gov.pl (K.S.);; 2 Department of Physical Chemistry and Technology of Polymers, Silesian University of Technology, 44-100 Gliwice, Poland; 3 Department of Engineering Materials and Biomaterials, Silesian University of Technology, 44-100 Gliwice, Polandjakupol064@student.polsl.pl (J.P.); 4 Materials Research Laboratory, Silesian University of Technology, 44-100 Gliwice, Poland

**Keywords:** PBX, silicone rubbers, energetic materials, explosive hardening

## Abstract

Modern energetic materials (EMs) have many different civil applications. One of their most promising applications in civil engineering is explosive hardening, which facilitates the fast and cost-effective improvement of mechanical properties in the treated material. In this work, we present the results of our investigation on the explosive hardening of S235JR Steel with PBX formulations containing silicone binders and 1,3,5-trinitro-1,3,5-triazinane (RDX). In terms of safety, the impact (5–15 J) and friction (240–360 N) sensitivity of the tested plastic-bonded explosives (PBXs) was verified, simultaneously with DSC tests, energy of activation calculations, and critical diameter measurement. The developed material, prepared with techniques similar to the anticipated working conditions, is characterized by a high detonation velocity (up to 7300 m/s), low sensitivity for mechanical factors (10 J, 288 N), and a small critical diameter (3.3 mm). The developed PBX based on a silicone binder demonstrated grain fragmentation, recrystallization, and an increase in the surface hardness of S235JR steel, which was confirmed with SEM, EBSD, microstructure analysis, and microhardness studies.

## 1. Introduction

Explosive hardening is commonly employed in order to modify the mechanical properties of a variety of alloys, primarily affecting their hardness, plasticity, and strength [1,2,3]. Hadfield (high-manganese) steels are a common subject for this type of hardening, due to their high impact strength and resistance to abrasion in the hardened state, which are highly desirable for a variety of applications [4]. That said, many other metals and alloys, e.g., titanium, can be explosively hardened to tailor their properties to a variety of applications [5,6,7]. An important and under-researched aspect of the state of the art is the potential of explosive hardening methods in fine-tuning the properties of low-manganese structural steels.

A number of different sources of shock waves have been used in explosive hardening, such as ammonals, emulsion explosives, plastic-bonded explosives (PBXs), and traditional energetic materials (EMs) [6]. In terms of energetic material selection, PBXs appear to be the most favourable choice, as the other EMs are adversely affected by low temperatures, becoming brittle and less susceptible to initiation. The important advantages of PBXs in this application is their high uniformity and facile processing, resulting in highly homogeneous shock wave parameters being achieved across the entire hardened surface [8].

In terms of PBX design, a number of different binders are commonly used, including polystyrene, polyisobutylene, and polyurethane rubbers [9,10]. Fewer works have been reported on the use of silicone rubbers as binders for PBX formulations, even though they afford the PBXs a number of potentially valuable features and properties [11,12]. This ties into the trend of utilising polysiloxanes and their derivatives in EM formulations, which has been the subject of a recent review [13].

Silicone rubbers are polysiloxane elastomers with a general repeat unit formula of R_2_SiO. They are typically produced via the hydrolysis of chlorosilanes and the subsequent polymerisation/polycondensation. Silicone rubbers are known for their high thermal and chemical resistance (particularly to oils, in contrast to classical rubbers), relatively high thermal conductivity, and high flexibility [14]. Due to these features, silicone rubbers have found extensive application in the automotive industry, biomiedical applications, and aviation and aerospace industry, as well as in the manufacture of electronics [15]. Even though silicone rubbers have been studied in several applications related to PBXs (as mentioned above), they do not appear to have been used in the context of applying PBXs to the explosive hardening of steel.

In light of the above, in this work, we have focused our study on the use of RDX-based PBX formulations in the explosive hardening of the common S235JR construction steel, being the first literature report to use PBXs containing silicone rubbers for the explosive hardening of steel. The two types PBX formulations differed in terms of the utilised silicone rubber and were further differentiated by the amount of the silicone binder used. The goal of this research was to evaluate how the properties and amount of the binder affect the key performance benchmarks of the PBX formulations, as well as to provide an initial outlook on how these benchmarks translate to the quality of the explosive hardening process and its products. Taking into account that most explosive hardening procedures utilise EMs that are produced on-site, we have opted to recreate these conditions as faithfully as possible and limited charge preparation procedures to what is feasible on-site, hence, e.g., vacuum elaboration techniques were omitted.

## 2. Materials and Methods

### 2.1. Materials

The RDX (Class 5 according to the MIL-DTL-398D standard) was produced by Nitrochem S.A. (Bydgoszcz, Poland), and silicone rubbers Ecoflex 00-30 and Dragon Skin 30 were commercially obtained from Kauposil Sp z.o.o (Siemianowice Śląskie, Poland). All materials were used as received, without any purification.

### 2.2. Preparation of PBX Samples

The pre-weighed portions of RDX were dried at a temperature of 60 °C for 12 h. The mass of the RDX sample was in all cases suitable to prepare 1 kg batch of corresponding PBX. For each composition, the preparation procedure was the same. The silicone elastomer (component A) and silicone elastomer cross-linking agent (component B) of each silicone rubber were weighed and transferred into the mixing chamber of a DRAIS-type mixing machine (20–40 rpm). Component A and component B were used in a weight ratio of 1:1. In the next step, the components were blended for 5 min in mixer at a speed of 20–40 rpm. After that, the mixed silicone polymer had been transferred into a vacuum vessel and was degassed for 5 min under a 0.2 bar pressure. After degasification, subsequent portions of RDX (50 g each in 1 min intervals) were added to the silicone matrix with constant mixing (20–40 rpm). After the addition of the last RDX portion, the whole composition was mixed for another 5 min. The compositions of the PBX formulations are summarised in Table 1. The prepared PBXs were cast into 3D-printed cases to cure for 24 h at 23 °C.

### 2.3. Performance Testing

#### 2.3.1. Differential Scanning Calorimetry (DSC)

A Mettler Toledo DSC 3 instrument was used for studying the thermal exothermic decomposition processes of PBX samples. The measurements of each samples were carried out for approximately 1 ± 0.1 mg of the sample placed in a sealed aluminium crucible. In order to derive the kinetic information, the samples were heated from 30 to 300 °C, using heating rates (β) of 3, 5, 7, and 10 °C/min, respectively.

The activation energy for the thermal decomposition reaction of the PBX formulations was calculated using the Kissinger and Ozawa methods [16]. The Kissinger method describes the following equation:

(1) $$ln\frac{\beta }{T_{p}^{2}}=ln\left(\frac{AR}{E_{k}}\right)-\left(\frac{E_{k}}{RT_{p}}\right)$$

where β is the heating rate, *T_p_* is the maximum temperature peak for a given β, *A* is the pre-exponential factor, *R* is gas constant, and *E_k_* is the energy of activation determined by the Kissinger method. The activation energy can be calculated from the slope of the line in the plot *ln*β/*T_p_^2^* vs. *1/T_p_*, which multiplies by the *R* constant.

The Ozawa method describes the following equation:

(2) $$log\left(\beta \right)=log\left(\frac{AE_{o}}{RG(\alpha )}\right)-2.315-0.4567\frac{E_{o}}{RT_{p}}$$

where α is the extent of conversion and *E_o_* is the energy of activation determined by the Ozawa method.

#### 2.3.2. Sensitivity Testing

The friction sensitivity was determined using a Peters apparatus according to the standard [17]. A thin layer of PBX sample was placed on the surface of the porcelain plate. Changes in the normal force between the porcelain pistil and the plate were achieved by applying different loads. The sample initiation was observed through sound, smoke appearance, or by the characteristic smell of the decomposition products. The lowest normal force value at which the signs of initiation were noticeable in at least one of the six trials is reported as the friction sensitivity.

The impact sensitivity was determined using a BAM Fallhammer apparatus according to the standard [18]. The impact sensitivity of PBX samples was evaluated using a Kast fall hammer instrument. For this test, 40 mm^3^ of each sample was placed between two metallic cylinders fixed in a bigger metallic ring. The lowest impact sensitivity was evaluated as the energy necessary to initiate (flame, crackle, smoke, and smell) of the sample in at least one of six trials.

#### 2.3.3. Critical Diameter Measurement

**Figure 1:**
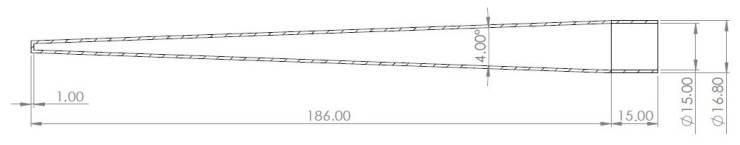
Scheme of the conical charge casings used for critical diameter measurements.

In order to determinate the critical diameter of the tested PBXs, the cones were prepared using of a Prusa Mk3 3D printer (Prusa Research, Prague, Czech Republic) and Noctuo PETG filament (Noctuo Sp. Z.o.o., Gliwice, Poland). The geometry of the cones used in this study is presented in Figure 1. The elaborations were performed with small doses of PBX until the maximum density was reached. After this process, the charges were maintained in stable conditions to provide full curing (24 h, 23 °C).

Instead of a booster, after the elaboration process, each cone was closed with a push pin, which allowed detonator placement 5 mm deep inside the cylindrical part of the cone. Each charge was connected with a witness plate (S215 steel, 200 × 100 × 1 mm), placed on the base steel plate (S215JR steel 30 × 200 × 200 mm) and connected to a NITRODET 0.2A detonator. (Nitroerg S.A., Bieruń, Poland) After detonation, based on the witness plate deformation, the distance of the detonation was measured with a calliper. Based on this parameter and the geometry of the cone, the critical diameter was calculated.

### 2.4. Explosive Hardening

#### 2.4.1. Tested Materials

The explosive hardening process was performed on two sheets of unalloyed structural steel S235JR with the chemical composition given in Table 2. Samples shaped like circles (Sheet 1, d = 50 mm) and squares (Sheet 2, 50 × 50 mm) were taken from a 150 × 150 × 10 mm sheet material. The material was tested after explosive hardening with the use of E3RDX8 charges (Table 3). The sheets was tested with an additional technological spacer in the form of a 1 mm-thick layer PETG.

#### 2.4.2. Explosive Hardening System

**Figure 2:**
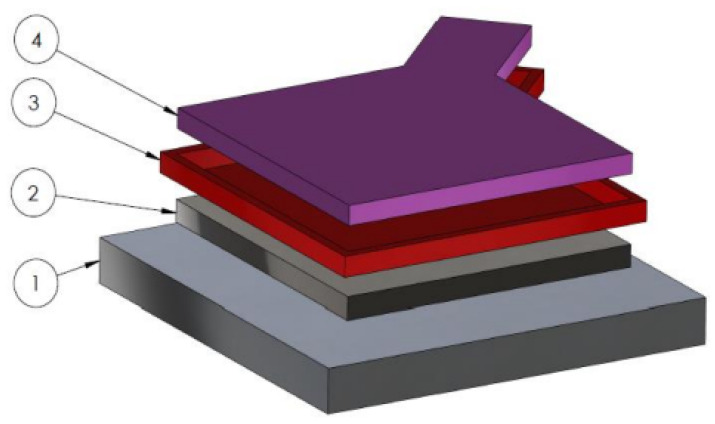
Technological system for the explosive hardening of steel, where 1—base plate; 2—hardened steel plate; 3—3D printed PETG mould for the PBX sheet; 4—E3RDX8 sheet.

Prior to the explosive hardening process, the base steel plate and hardened steel plate (1 and 2 in Figure 2) were subjected to mechanical cleaning by grinding and chemical cleaning, following a multi-step washing and degreasing process. In the next step, the tested sheets were placed centrally on a base steel plate (designed to decelerate the movement of the explosion-driven hardening sheet and to level the system). The moulds (3 in Figure 2), used for elaborating the E3RDX8, were prepared using the 3D-FDM printing technique with the Noctuo PETG filament (Noctuo Sp. Z.o.o., Gliwice, Poland). In the final stage of the preparation, the moulds filled with E3RDX8 were deposited on the surface of the processed sheets (4 in Figure 2). After completing the preparation of the test system, E3RDX8 charges were initiated using NITRODET 0.2A detonators. In the process of carrying out the surface modification of S235JR steel, the velocity of detonation (VoD) of the charges was measured using the short-circuit method. VoD data were recorded using an AMC VIBRO CONDITION 8000D (AMC VIBRO Sp. z o.o., Kraków, Poland) signal conditioner and a Tektronix TBS2401B (Tektronix Inc., Beaverton, OR, USA) digital oscilloscope. The explosive hardening setup is shown in Figure 2.

After explosive hardening, the sheets were mechanically cleaned and sent for further processing. The sheets were cut with a resistance wire under intensive cooling to circle (diameter equal to 50 mm)- and square (50 × 50 mm)-shaped samples. In order to obtain a brittle fracture of the hardened steel, the samples were cooled in liquid nitrogen and mechanically broken. The samples for EBSD and microhardness testing were subjected to mechanical cleaning processes by grinding and then polishing.

#### 2.4.3. Observation of the Fracture Structure and Surface Topography of Blast-Hardened S235JR Steel Using Scanning Electron Microscopy

The observation of the structure and topography of S235JR steel was performed using a Zeiss FEG SEM Supra 35 scanning electron microscope. Images were obtained using the SE (Secondary Electron) detection method at an accelerating voltage of 20 kV. The chemical composition of the samples was also analysed using an EDS X-ray energy spectrometer from Thermo Scientific, which is part of the microscope instrumentation.

#### 2.4.4. Analysis of the Crystallographic Orientation of Grains and Phases Using the EBSD Backscattered Electron Diffraction Method

The crystallographic grain orientation analysis and phase identification of S235JR steel was performed with a Zeiss FEG SEM Supra 35 electron microscope (Carl Zeiss NTS GmbH, Oberkochen, Germany) using an EDAX backscattered electron diffraction detector included in the instrumentation.

#### 2.4.5. Vickers Microhardness Test

The Vickers microhardness test was carried out on a Future-Tech FM-ARS 9000 (Future-Tech, Kanagawa, Japan) on the surface of the samples under a load of 2.942 N. The duration of the loading force was 15 s. The measuring point is shown in Figure A15.

## 3. Results and Discussion

### 3.1. Differential Scanning Calorimetry

**Figure A1:**
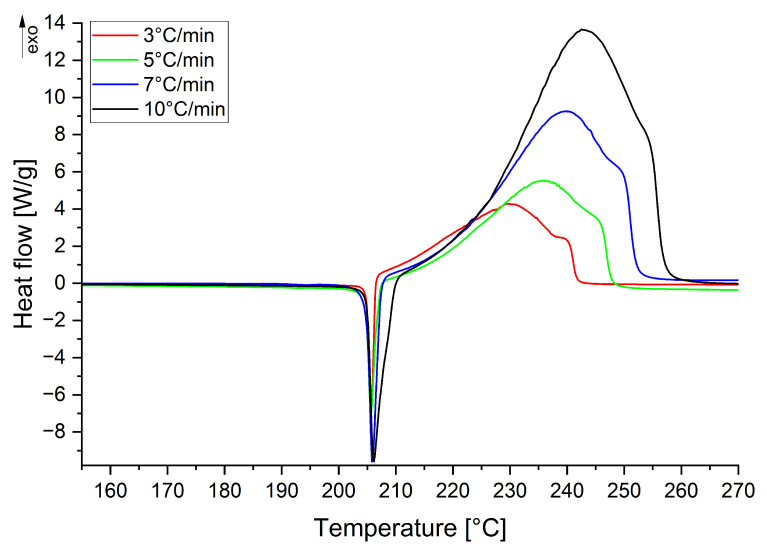
DSC curves of RDX at heating rates equl to 3, 5, 7, and 10 °C/min.

**Figure A2:**
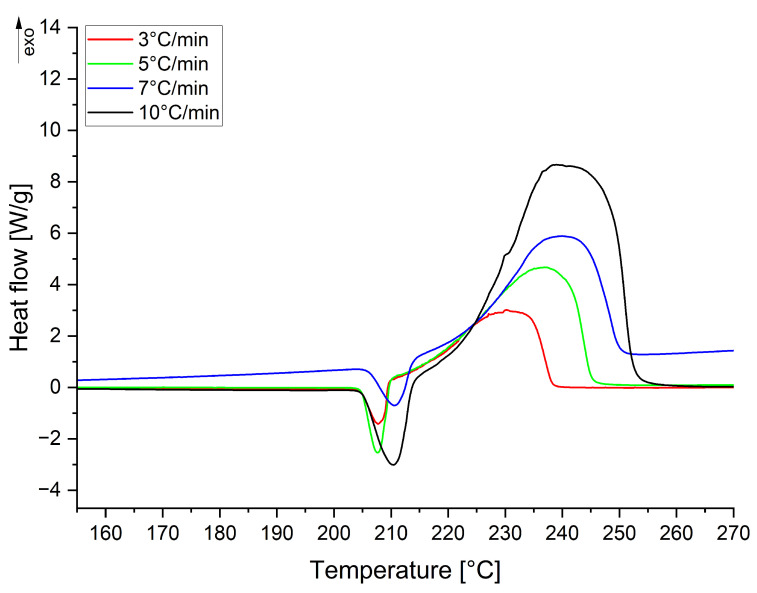
DSC curves of E3RDX7 at heating rates equl to 3, 5, 7, and 10 °C/min.

**Figure A3:**
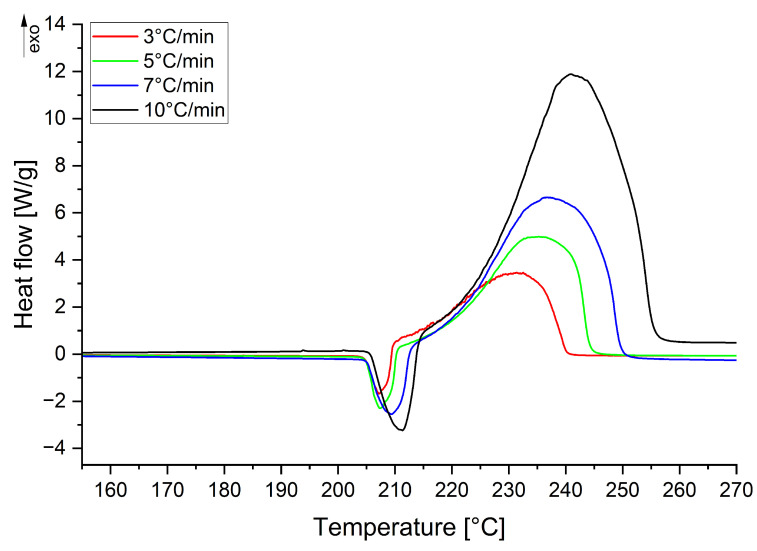
DSC curves of E3RDX8 at heating rates equl to 3, 5, 7, and 10 °C/min.

**Figure A4:**
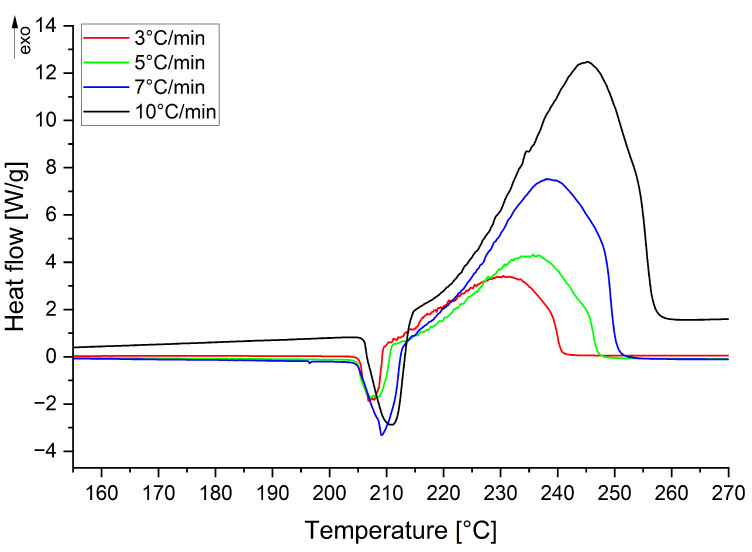
DSC curves of E3RDX9 at heating rates equl to 3, 5, 7, and 10 °C/min.

**Figure A5:**
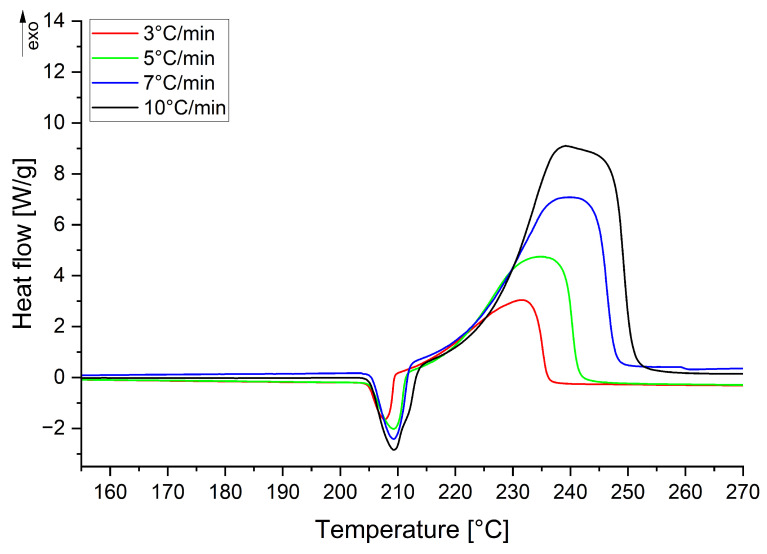
DSC curves of DS3RDX7 at heating rates equl to 3, 5, 7, and 10 °C/min.

**Figure A6:**
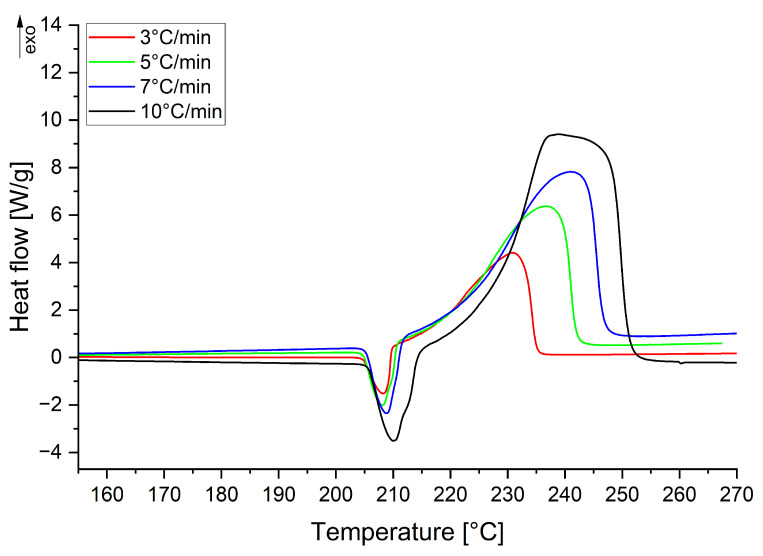
DSC curves of DS3RDX8 at heating rates equl to 3, 5, 7, and 10 °C/min.

**Figure A7:**
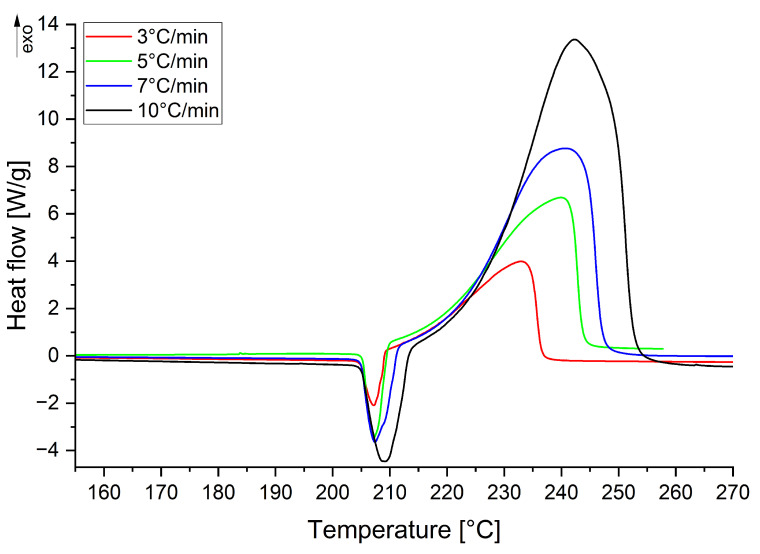
DSC curves of DS3RDX9 at heating rates equl to 3, 5, 7, and 10 °C/min.

The analysis of the thermal decomposition of RDX and its formulations with silicone rubbers was conducted. DSC curves of RDX- and RDX-based polymer-bonded explosives can be seen in Figure A1, Figure A2, Figure A3, Figure A4, Figure A5, Figure A6 and Figure A7. The enthalpy ($\Delta H_{p}$) and peak temperatures ($T_{p}$) of the exothermic reactions of the PBX formulations for different heating rates are listed in Table 4 and Table 5.

In Figure A1, the DSC thermograms of RDX, recorded at heating rates of 3, 5, 7, and 10 °C/min, all show a sharp endothermic peak between 200 and 210 °C, subsequently following a broad exothermic peak between 220 and 260 °C [19]. For both PBX samples using Ecoflex 00-30 and Dragon Skin 30 (Figure A2, Figure A3, Figure A4, Figure A5, Figure A6 and Figure A7) as binders, an endothermic peak appears around 210 ° C, followed by an exothermic peak between 220 and 260 °C. The addition of silicone rubbers seems to have no significant effect on the decomposition reaction of RDX.

Comparing the decomposition temperatures of pure RDX and of the PBX compositions, recorded at heating rates of 5, 7, and 10 °C/min, a slight shift towards lower temperatures can be observed for the PBXs. This suggests that both the Ecoflex 00-30 and Dragon Skin 30 binders accelerate the thermal decomposition of RDX.

**Figure 3:**
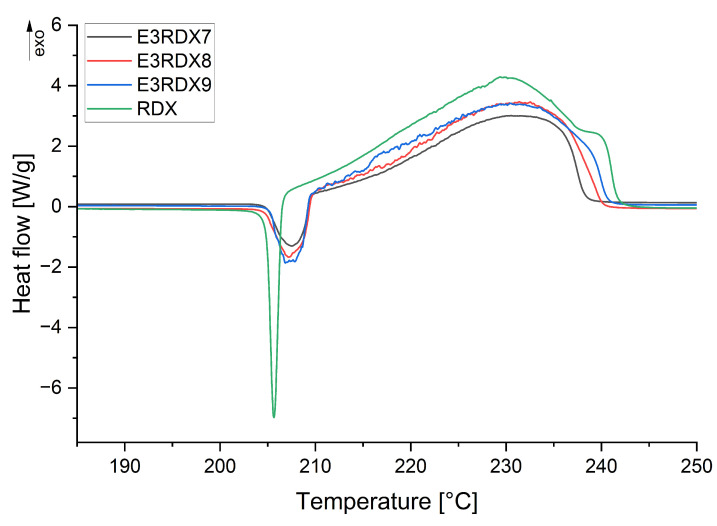
Effects of Ecoflex 00-30 content in PBX on the thermal decomposition of RDX at a heating rate equal to 3 °C/min by DSC.

**Figure 4:**
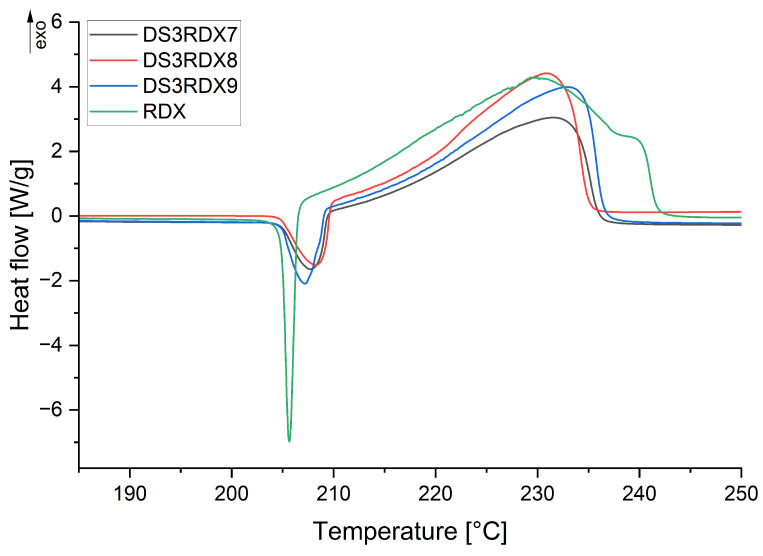
Effects of Dragon Skin 30 content in PBX on the thermal decomposition of RDX at a heating rate equal to 3 °C/min by DSC.

Therefore, to accurately assess whether the applied silicone rubbers are compatible with RDX, DSC curves were compared at a heating rate of 3 ° C/min, as presented in Figure 3 and Figure 4. The lowest heating rate was selected because it introduces the least thermal inertia [20], and the NATO Standardization Agency recommends determining the compatibility at a heating rate of 2 °C/min [21].

According to the STANAG 4147 standard [22], a mixture is classified as compatible if the difference in the exothermic peak temperature ($\Delta T_{p}$) ≤ 4 °C; incompatible if the $\Delta T_{p}$≥ 20 °C; and if the $\Delta T_{p}$ is between 4 and 20 °C, an alternative method for compatibility assessment is recommended. Regardless of whether the binder is Ecoflex 00-30 or Dragon Skin 30, the decomposition temperature is higher than that of pure RDX. The highest decomposition temperature difference ($\Delta T_{p}$ = 4 °C) between RDX and PBX at a heating rate of 3 °C/min was recorded for the DS3RDX9 sample, indicating that the applied silicone rubbers are compatible with RDX.

The apparent activation energies of the thermal decomposition reaction calculated by the Kissinger and Ozawa–Flynn methods [16], described by Equations (1) and (2), are presented in Table 6. The values of the apparent activation energy obtained by both methods are consistent, with minor differences between them, and the coefficient of determination (*R*^2^) values are generally high (>0.96), confirming good fit quality.

Among the tested PBXs, DS3RDX7 exhibits the highest apparent activation energy (304 kJ/mol by the Kissinger method and 297 kJ/mol by the Ozawa method), while E3RDX9 shows one of the lowest values (171 and 170 kJ/mol, respectively). The addition of both the Ecoflex 00-30 and Dragon Skin 30 (Kauposil Sp z.o.o, Siemianowice Śląskie, Poland) binders results in higher apparent activation energies compared to raw RDX, indicating an improvement in the thermal stability of RDX. The only composition exhibiting a slightly lower *E^k^* (by 2 kJ/mol) and *E*_o_ (by 3 kJ/mol) than pure RDX is E3RDX9. However, this difference is minor and may be attributed to the degree of PBX homogenization, which could have influenced the result for small sample used in tests (approximately 1 mg).

Comparing Ecoflex 00-30-based PBXs to Dragon Skin-based PBXs, the first show apparent activation energies significantly lower than Dragon Skin 30-based PBXs, suggesting that the Dragon Skin 30 binder enhances the thermal stability of PBX formulations more effectively than the Ecoflex 00-30 binder. This may be related to the fact that the cross-linked silicone rubber Dragon Skin 30, according to the manufacturer’s data [23], has a Shore A hardness of 30, while the cross-linked Ecoflex 00-30 is significantly softer, with a Shore 00-30 hardness [24]. The stiffer polymer matrix likely acts as a more effective physical barrier to the diffusion of heat and decomposition products, thereby requiring more energy to initiate thermal decomposition.

To sum up, the E3RDX9 sample exhibits a similar activation energy to that of pure RDX, which indicates that the stabilising effect postulated for silicone rubber did not occur. While this can be attributed to issues with the homogeneity of the PBX (due to the PBX consisting of 90% RDX), the issue is likely more complex. For both binders, the highest activation energy was observed at 70% RDX content (E3RDX7 and DS3RDX7 samples). Increasing the RDX content resulted in the decrease of the activation energy by approx. 50–60 kJ/mol. This decrease in activation energy occurs earlier for Dragon Skin (at only 80% RDX content) than for Ecoflex (at 90% RDX content). This corresponds to the higher viscosity and rigidity of Dragon Skin compared to Ecoflex. Consequently, the observed change in the activation energy of the PBX is likely due to the threshold ability of the silicone binders to effectively coat the RDX particles being exceeded in the PBX formulations containing 80% or more RDX. This firstly manifests as lesser stabilisation (i.e., activation energy is higher than that of pure RDX, but lower for the samples containing 70% RDX), and afterwards, as loss of stabilisation, as seen for the E3RDX9 sample.

### 3.2. Sensitivity Testing

The friction and impact sensitivity results are presented below (Table 7).

The results of the friction sensitivity tests clearly indicate that the type of binder used has a significant effect on the resistance of PBX formulations to the initiation of a energetic reaction with mechanical stimuli. The highest friction resistance was demonstrated by sample DS3RDX7, whose initiation threshold exceeded 360 N, making it the most resistant of all PBX formulations tested. In contrast, the E3RDX9 sample showed the least resistance, reaching a sensitivity of friction equal to 192 N—a value higher than that of pure RDX (144 N). Samples E3RDX7 and E3RDX8 also showed decreased sensitivity (288 N), while the other samples—DS3RDX8 and DS3RDX9—were within the range of 240 N, confirming the general trend of improved friction insensitivity as the mass proportion of binder in the composition increases.

It is worth noting that the mechanical properties of the binder play a crucial role in mitigating hotspot formation during frictional loading. Hard silicone rubber, such as Dragon Skin 30, is more resistant to local deformation and shear, reducing stress concentration at contact asperities and thereby increasing the friction ignition threshold [25]. In contrast, a softer binder like Ecoflex 00-30 may allow for greater localized heating due to the easier mechanical deformation, promoting ignition under lower loads [26]. Moreover, the E3RDX samples exhibited lower apparent activation energies than the DS3RDX samples, indicating reduced thermal stability. The particularly high friction sensitivity of E3RDX9 may also be related to poor homogenization, thus increasing the likelihood of the structural inhomogeneities that facilitate ignition.

As shown in Table 7, compositions E3RDX7 and E3RDX8 showed the highest impact resistance (15 J and 10 J, respectively), containing the Ecoflex 00-30 binder. In comparison, the DS3RDX8 and DS3RDX9 compositions using the tougher Dragon Skin 30 binder exhibited high impact sensitivity (5 J), comparable to that of pure RDX. It has been observed that for PBXs, when the content of the binder in the composition increases, the sensitivity to impact decreases. The opposite trend can be observed for samples containing Dragon Skin 30 as a binder, where the increased amount of binder does not reduce the impact sensitivity. Although Dragon Skin 30 provides better resistance to friction-induced ignition, its increased stiffness most likely leads to the weaker damping of the mechanical energy on impact, resulting in a greater load transfer directly to the RDX crystals and increasing the risk of their damage and the formation of local hotspots. The impact sensitivity results obtained could also be possibly related to the standard testing methodology adopted, in which the hammer impact leads to the adiabatic compression of the air in the sample chamber—a phenomenon recently highlighted in the literature [27].

The results of the impact sensitivity tests indicate the hardness of the silicone rubber used as a binder has a significant effect on the response of the PBX to mechanical stimuli. In contrast to the frictional mechanism, where harder matrices reduce local deformation and the likelihood of hotspot formation, impact effects are dominated by the fracture, micro-tensioning, and local heat accumulation within the material. Wickham et al. [28] showed that the elastic modulus of the binder has the greatest impact on the change in impact sensitivity. Their research confirms that increased matrix stiffness leads to a reduced ability to absorb impact energy and increases local stresses, promoting initiation. In the case of the DS3RDX sample, the stiffer Dragon Skin 30 does not provide effective energy damping, and the impact forces are directly transferred to the RDX crystals. The work of Dandekar et al. [29], on the other hand, indicates that the strength of adhesion between the binder and the EM particle affects heat generation through friction and fracture. In compositions with weaker adhesion (e.g., insufficient homogenization or chemical incompatibility), more pronounced decohesion is observed, which increases local heating. The E3RDX9 sample, which exhibits the highest impact sensitivity among PBXs from Ecoflex 00-30, may suffer from such a problem.

Considering the results of sensitivity to mechanical stimuli, PBX formulations such as DS3RDX7, E3RDX7, and E3RDX8 can be considered safe for use. Although the harder binder, Dragon Skin 30, improves PBX’s resistance to initiation through friction, it does not necessarily provide better impact protection. The softer Ecoflex 00-30 binder, with its higher elasticity and ability to dampen deformation energy, better absorbs impacts and protects the material structure from microcracks and hotspots.

### 3.3. Critical Diameter

The results obtained from the tests are summarised in Table 8 below.

All of the tested PBXs are characterized by a low critical diameter. The decrease in the measured value is in line with the increased content of EM in the composition with the expected effect of improved detonation parameters. The better results for PBXs based on Ecoflex 00-30 silicone rubber most likely stem from the lower initial viscosity (3000 mPa·s for Ecoflex 00-30 and 20,000 mPa·s for Dragon Skin 30), which facilitated a higher level of homogenisation and the more equal distribution of EM inside the polymer matrix. The low value of charge density is derived from the elaboration method, which was performed via cast-curing under atmospheric pressure. Without reduced pressure conditions, the shape of covers could strongly affect the elaboration process by allowing air bubble formation inside the charges.

### 3.4. Structure and Topography Analysis

**Figure 5:**
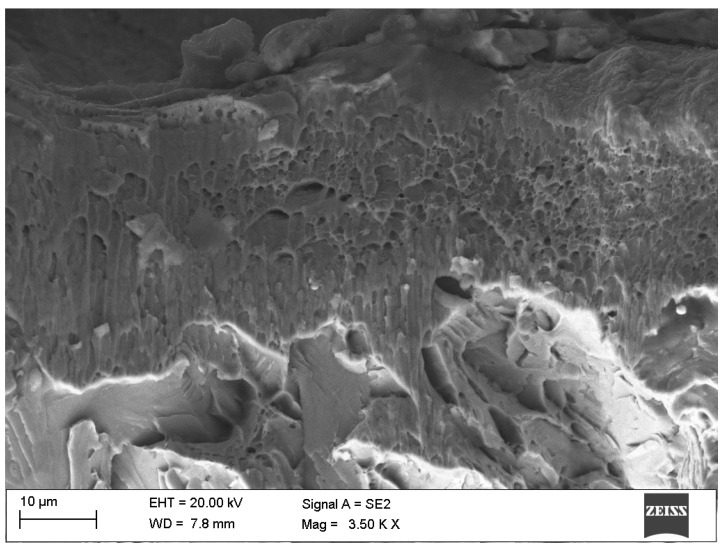
Fracture surface of S235JR steel after the detonation hardening process.

**Figure A8:**
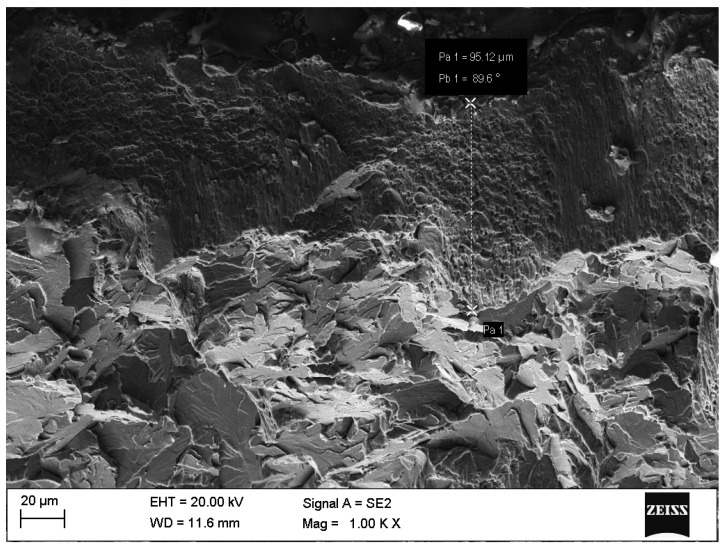
Fracture surface of S235JR steel with visible detonation hardening effects and the thickness of the strengthened surface.

**Figure A9:**
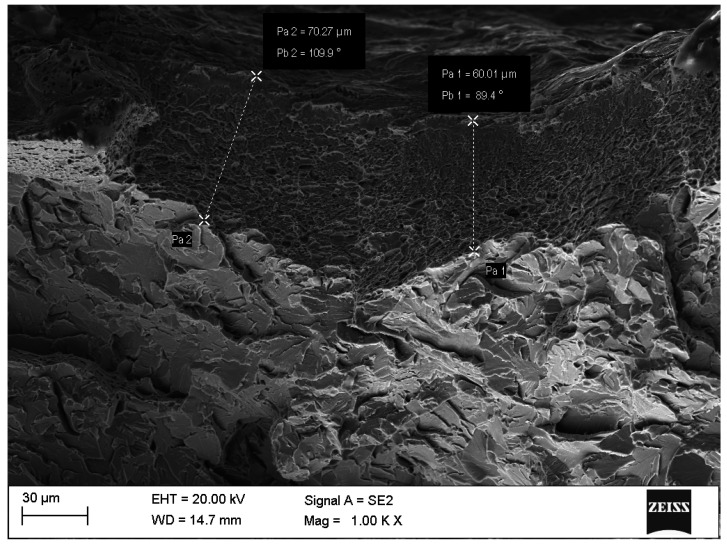
Breakthrough surface of S235JR steel with visible detonation hardening effects and the thickness of the strengthened surface.

**Figure A10:**
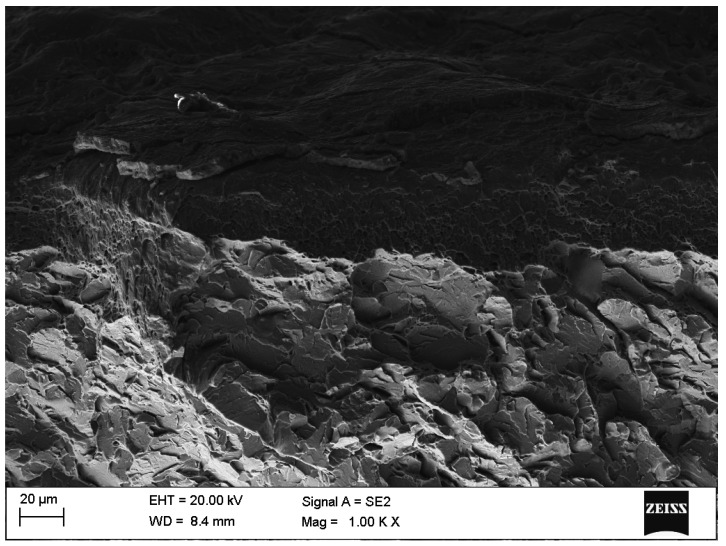
Fracture surface of S235JR steel after detonation hardening process with visible surface detachment.

**Figure A11:**
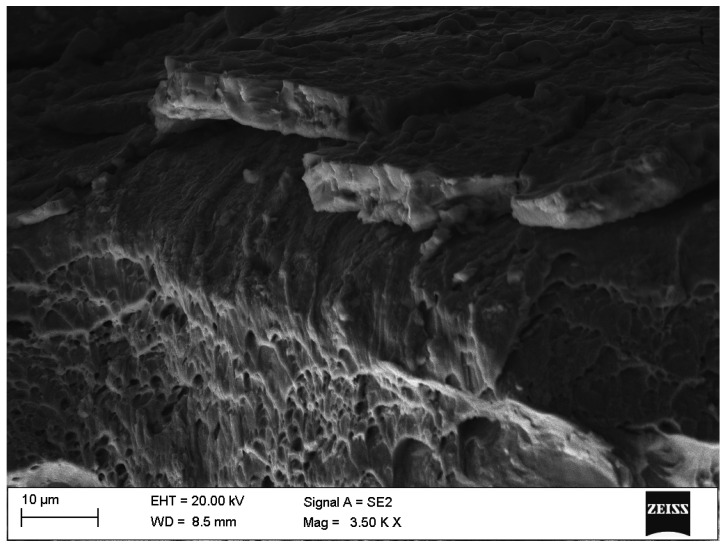
Fracture surface of S235JR steel after detonation hardening process with visible surface detachment.

Based on scanning electron microscope studies of the structure and surface topography of the tested samples, it can be confirmed that a hardened surface layer of S235JR steel was obtained. The hardened layer produced is characterized by a relatively uniform thickness over the entire surface of the specimen (Figure A8 and Figure A9). The surface topography of the S235JR steel tested is characterized by the presence of spherical impurities, significantly different from the sample proper (Figure 5). These impurities are most likely due to the rapid solidification of the material consumed during sample preparation, i.e., the resistance wire that was used to cut the test specimens. Furthermore, visible pores and voids result from the material displacement during rapid deformation, as well as secondary recrystallization. In Figure A10 and Figure A11, the partial detachment of the surface of the S235JR steel caused by the rapid passage of the shock wave can be seen.

### 3.5. Analysis of the Crystallographic Orientation of Grains and Phases

**Figure 6:**
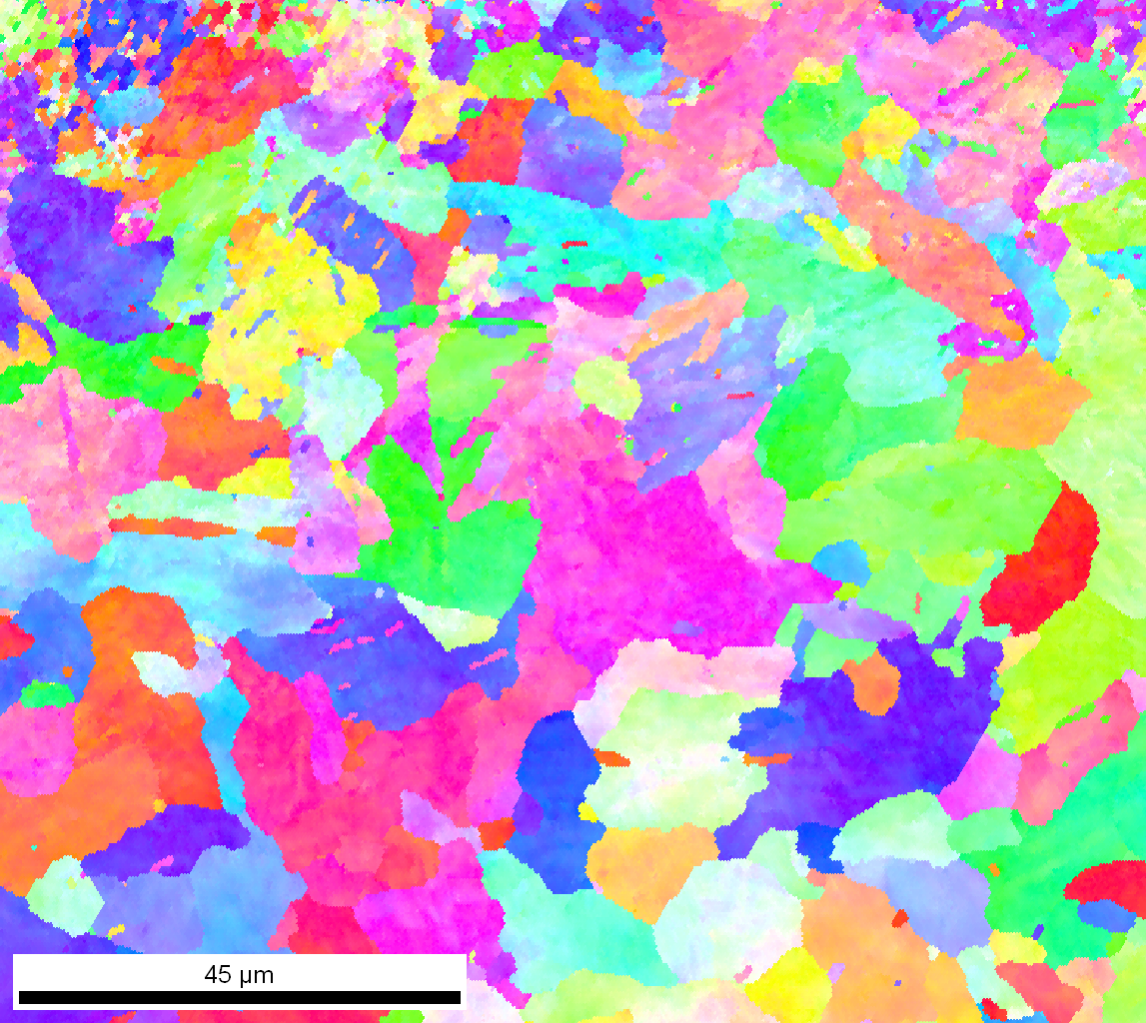
EBSD map of the grain crystallographic orientation (Inverse Pole Figure map—colours indicate the crystal direction).

**Figure A12:**
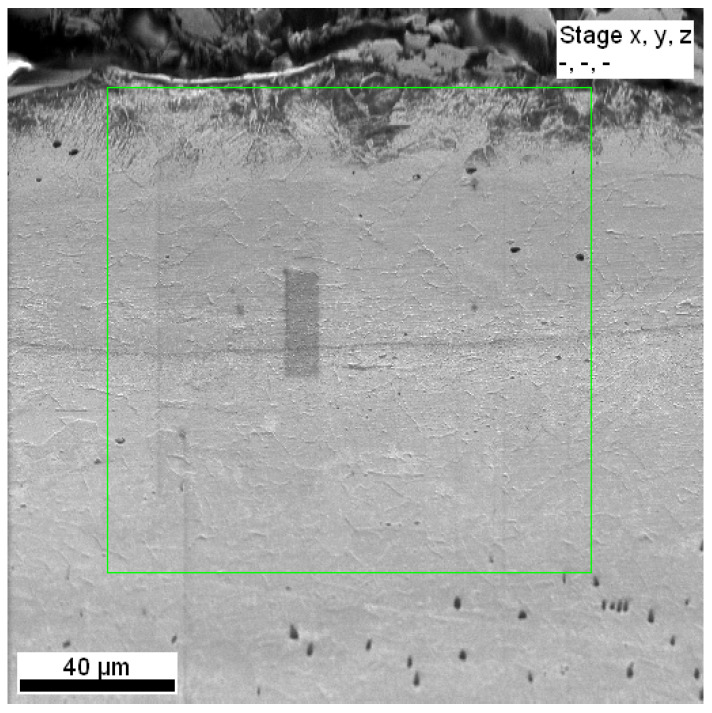
Structure of hardened S235JR steel with the EBSD analysis micro-area marked.

**Figure A13:**
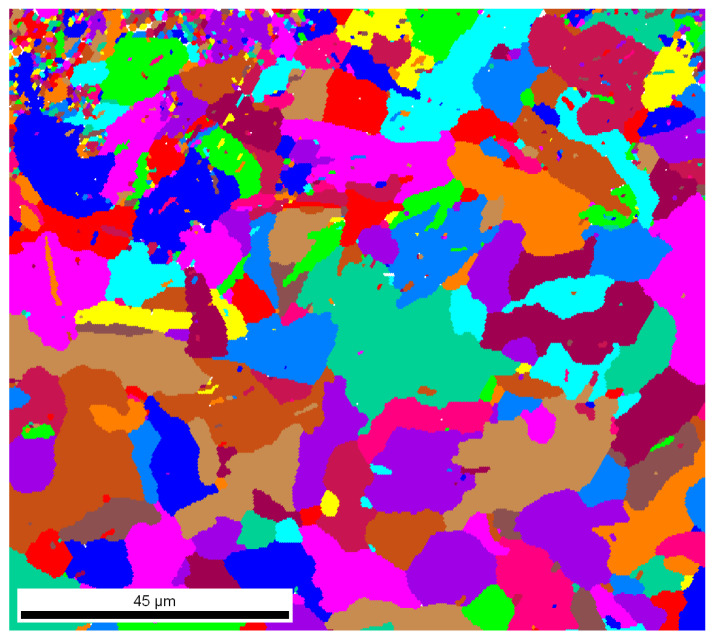
EBSD map of the grain size (colours indicate the different grains).

**Figure A14:**
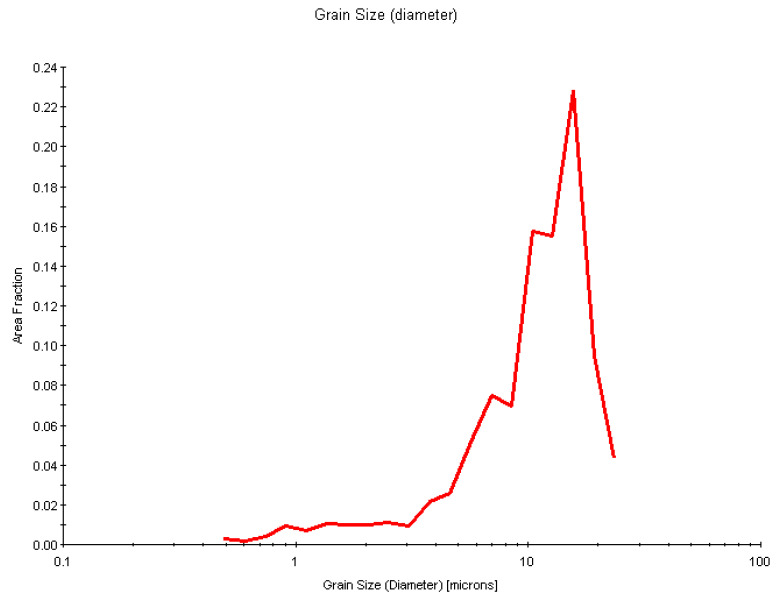
Graph of grain size distribution of S235JR steel after detonation hardening.

An analysis of the crystallographic orientation of the grains and the identification of the phases of the steel tested was carried out using the EBSD method. The area of the sample on the surface of which the analysis was carried out can be seen in Figure A12. Figure 6 shows the crystallographic orientation of the ferrite grains, and the grain size map obtained (Figure A13), from which the fragmentation of the grains, especially in the surface layer, was confirmed. A grain size gradient is evident where the smallest grains are in the near-surface zone of the material, while their size increases with depth into the material. The pronounced fineness of the grains in the surface layer zone is likely to increase the mechanical properties, including the hardness. The resulting grains are characterized by a random orientation—this is due to the creation of violent stresses in the material and the high kinetic energy delivered during detonation. The presence of such fine grains with a random orientation is typical of detonation-hardened materials. The highest surface fraction is found for grains with a diameter of about 10 µm, where the surface fraction reaches a value close to 0.22 (Figure A14).

**Figure 7:**
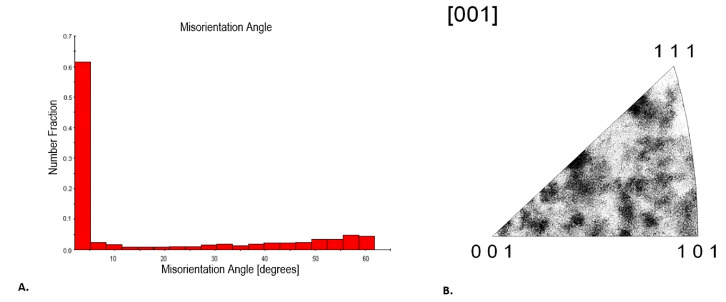
(**A**) Grain disorientation angle diagram of S235JR steel after detonation hardening. (**B**) Reverse polar figure of S235JR steel after detonation hardening.

This confirms the achievement of a fine crystalline structure in the surface layer of the modified steel. Figure 7 shows the obtained grain disorientation angle plot, where the value of the numerical fraction is highest for an angle close to 0°, indicating that most adjacent grains have a marginal disorientation angle. The fractions corresponding to low angles (up to about 10°) represent the highest percentage of occurrences, with a dominant value of about 0.7 for an angle close to 0°. For angles greater than 10°, the numerical fraction drops sharply and remains low. This distribution of disorientation angles suggests that the material is characterized by low disorientation angles between most adjacent grains. This implies that the steel has a structure with relatively ordered grain boundaries, where the grains are characterized by a similar crystallographic orientation. From the inverse polar figure obtained (Figure 7), the grains of the material are not completely randomly oriented. The absence of clear, very dark points at the [111], [101] and [001] vertices indicates that the steel does not have a strong directional texture but instead shows a diffuse crystallographic orientation. However, there are still areas of a slightly higher density, suggesting a low-intensity texture. This may indicate anisotropy in the properties of the detonation-hardened steel.

### 3.6. Vickers Microhardness Test

**Figure A15:**
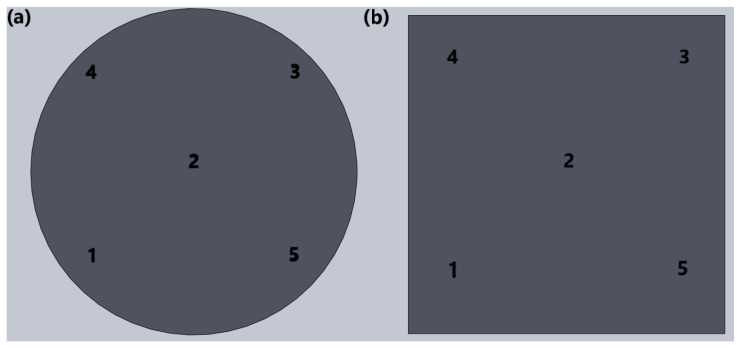
Diagram showing a (**a**) circle for the sheet 1 sample and (**b**) square for the sheet 2, together with the locations where microhardness measurements were taken. The numbers 1 to 5 indicate the locations and the sequence in which the microhardness measurements were performed.

The Vickers microhardness test was carried out on Future-Tech’s FM-ARS 9000 on the surface of the samples under a load of 2942 N. The duration of the loading force was 15 s. The hardness of S235JR steel not subjected to detonation hardening was 177 ± 6 HV. The specimens analysed had square and circular shapes, which consequently influenced the choice of measurement location (Figure A15). The measurement was carried out five times at each point. The square samples had dimensions of 50 × 50 mm, while the round samples had a diameter of 50 mm. The results shown in Table 9 confirm the increase in surface hardness of the detonation-hardened S235JR steel. The average value of the microhardness obtained for the treated steel is 242 HV, an increase in value of about 36% compared to the unhardened steel. The detonation hardening process increased the surface hardness of the steel and, consequently, increased mechanical the properties and wear resistance due to the comminution of grains in the surface layer of the modified component.

## 4. Conclusions

The purpose of this study was to investigate and evaluate RDX-based PBX compositions using two commercially available silicone rubbers—Ecoflex 00-30 and Dragon Skin 30—for their use in blasting operations, particularly in the context of the on-site preparation of PBXs for explosive hardening. A simplified elaboration method was deliberately used, without vacuum degassing or pressing, to replicate field conditions. The results provide valuable information on the performance, safety, and practical suitability of these PBX formulations.

Both series of PBX compositions, regardless of the silicone rubber used, were shown to have sufficient thermal stability and compatibility with RDX, as confirmed by the DSC analyses and activation energy calculations performed using the Kissinger and Ozawa methods. Nevertheless, significant differences were observed between compositions with different binders in terms of sensitivity to mechanical stimuli. Compositions containing Dragon Skin 30 showed lower friction sensitivity, which was attributed to the binder’s higher hardness and stiffness, reducing local deformation and hotspot formation. However, at the same time, these materials exhibited higher impact sensitivity, especially at a lower binder content (DS3RDX8 and DS3RDX9), which poses an increased risk during mixing and handling. This is likely due to the limited ability of the stiff binder to dissipate mechanical energy during dynamic loading, which is consistent with our current knowledge on the effect of matrix stiffness on PBX sensitivity. In contrast, compositions based on Ecoflex 00-30 showed lower critical diameters (as low as 2.5 mm for E3RDX9), indicating better detonation properties. This improvement may be the result of better homogenization, made possible by the much lower viscosity of Ecoflex than Dragon Skin 30 during mixing, which allowed for a more uniform distribution of EM in the polymer matrix. In addition, PBXs with Ecoflex 00-30 showed a higher impact resistance and acceptable friction sensitivity, especially in the E3RDX7 and E3RDX8 samples.

The results confirmed that both PBX compositions can be effectively cross-linked under ambient conditions to form durable and stable charges. This opens up the possibility of preparing them directly at the blasting site, in various geometric forms. However, special attention should be paid to the geometry of the components—for larger thicknesses or volumes, vacuum or pressing may be necessary to avoid gas voids and achieve a uniform density. Explosive hardening tests conducted with the E3RDX8 composition confirmed its effectiveness in the surface hardening of S235JR steel. Microstructural analyses by SEM and EBSD showed grain fragmentation, recrystallization, and an increase in surface hardness (up to 273.6 HV), confirming the effectiveness of the proposed material in practical applications.

In conclusion, it has been shown that PBX compositions based on silicone rubbers can be safely and effectively prepared and used in field conditions, and systems based on Ecoflex 00-30 offer the best compromise between safety, detonation properties, and ease of processing. In further studies, it is worth considering scaling up the method and using vacuum techniques to increase the homogeneity and reproducibility of the material parameters.

## 5. Future Outlook

The E3RDX8 composition can be identified as the most promising composition for future applications. This composition exhibited an optimal combination of moderate sensitivity (288 N, 10 J), a high stability (E_a_ in the range of 231–243 kJ/mol, compared to anE_a_ of 173 kJ/mol for RDX), and a low and repetitive critical diameter (3.3 ± 0.2 mm), as well as the desired energetic properties.

Future works should take into consideration the following issues:

- Maintaining the balance between the production/handling safety and initiation sensitivity of PBXs;
- The relationship between PBX’s layer thickness and hardened material properties and geometry;
- The PBX designed for application in explosive hardening ought to exhibit a short time and distance of detonation development and a low critical diameter/critical layer thickness;
- The process of preparing PBX should be relatively simple and fast, taking into account the viscosity of the composition to ensure the greatest possible degree of homogenization;
- The market demand for the development of more environmentally-friendly solutions needs to be taken into consideration in the development of future PBXs.

Due to extremely different properties of materials subjected to explosive hardening (e.g., sound speed in the material, tensile strength, hardness, microstructure, content), the issue of developing a versatile PBX charge is a complex challenge. The most likely solution would be to elaborate a series of PBXs, prepared from the same components but varying in terms of EM content. In this manner, it would be feasible to create a group of 3–5 PBXs that exhibit similar mechanical properties and processing conditions but vary in detonation properties (especially in VoD and detonation pressure); meanwhile, the dependence between PBX VoD (which is directly connected with detonation pressure) and sound speed in processed materials is an extremely important parameter affecting the whole process’ feasibility.

Due to its nature, the explosive hardening process can lead to the presence of internal stresses in the processed materials. Future works ought to address the issue of post processing on the properties of hardened materials. To exemplify this, a thermal relaxation process may be employed to reduce the stresses and influence the crystallographic structure of the material, giving more opportunities to fine-tune the material properties. Moreover, a thermal relaxation process can allow the improvement of the mechanical properties of the material (e.g., reduced crack propagation probability, which enhances the material durability).

The prospective applications of explosive hardening may include the production of lightweight elements for the aeronautic and space industry (e.g., elements of wings, fins, combustion chambers, and jet engine parts), parts of composite (e.g., NERA) armours for vehicles, ship building, rail production, the production of tools for cavity processing (e.g., for lathing and milling), or in construction elements which are required to have high mechanical properties (e.g., in bridge constructions).

## Figures and Tables

**Table 1:** Compositions of the investigated PBXs.

Sample Name	RDX [wt.%]	Ecoflex 00-30 [wt.%]	Dragon Skin 30 [wt.%]
E3RDX7	70	30	-
E3RDX8	80	20	-
E3RDX9	90	10	-
DS3RDX7	70	-	30
DS3RDX8	80	-	20
DS3RDX9	90	-	10

**Table 2:** Chemical composition of S235JR structural steel.

Elements	Max. Value [wt.%]
C	0.17
Mn	1.40
P	0.035
S	0.035
N	0.012

**Table 3:** Characteristics of the E3RDX8 charges used in the explosive hardening process.

Sheet	Layer Thickness [mm]	Density [g/cm^3^]	Velocity of Detonation [m/s]
1	9	1.43	6900
2	9	1.50	7300

**Table 4:** Enthalpy and peak temperatures of the exothermic peak for E3RDX7, E3RDX8, E3RDX9, and RDX.

Heating Rate [°C/min]	E3RDX7		E3RDX8		E3RDX9		RDX	
	$\Delta H_{p}$ [J/g]	$T_{p}$ [°C]	$\Delta H_{p}$ [J/g]	$T_{p}$ [°C]	$\Delta H_{p}$ [J/g]	$T_{p}$ [°C]	$\Delta H_{p}$ [J/g]	$T_{p}$ [°C]
3	943	231	1193	232	1366	230	1592	230
5	1027	237	1091	236	1516	236	1537	236
7	772	239	1243	237	1419	238	1770	240
10	1113	241	1399	243	1416	245	1835	244

**Table 5:** Enthalpy and peak temperatures of exothermic peak for DS3RDX7, DS3RDX8, DS3RDX9, and RDX.

Heating Rate [°C/min]	DS3RDX7		DS3RDX8		DSRDX9		RDX	
	$\Delta H_{p}$ [J/g]	$T_{p}$ [°C]	$\Delta H_{p}$ [J/g]	$T_{p}$ [°C]	$\Delta H_{p}$ [J/g]	$T_{p}$ [°C]	$\Delta H_{p}$ [J/g]	$T_{p}$ [°C]
3	950	231	1050	232	1233	234	1592	230
5	954	237	1093	237	1280	240	1537	236
7	1023	239	1034	238	1359	241	1770	240
10	1057	241	1161	241	1480	243	1835	244

**Table 6:** Activation energies of the decomposition for the investigated PBXs, determined via the Kissinger and Ozawa methods.

Sample Name	*E_k_* [kJ/mol]	*R* ^2^	*E_o_* [kJ/mol]	*R* ^2^
E3RDX7	246	0.9715	242	0.9725
E3RDX8	234	0.9926	231	0.9931
E3RDX9	171	0.9777	170	0.9797
DS3RDX7	304	0.9934	297	0.9937
DS3RDX8	257	0.9629	253	0.9651
DS3RDX9	260	0.9872	256	0.9879
RDX	173	0.9986	173	0.9987

**Table 7:** Friction and impact sensitivity of tested PBXs.

Sample Name	Friction [N]	Impact [J]
E3RDX7	288	15
E3RDX8	288	10
E3RDX9	192	5
DS3RDX7	>360	10
DS3RDX8	240	5
DS3RDX9	240	5
RDX	144	5

**Table 8:** Critical diameter of PBX charges.

Sample Name	Density [g/cm^3^]	Critical Diameter [mm]
E3RDX7	0.38	3.6 ± 1.0
E3RDX8	0.35	3.3 ± 0.2
E3RDX9	0.33	2.5 ± 0.1
DS3RDX7	0.37	4.7 ± 0.1
DS3RDX8	0.35	4.0 ± 0.8
DS3RDX9	0.34	2.9 ± 0.9

**Table 9:** Results of the Vickers microhardness measurements of S235JR steel after detonation hardening.

Sheet	Shape of Sample	Average Microhardness [HV]
1	Circular	234.08
1	Circular	235.4
1	Circular	239.6
2	Square	242.25
2	Square	273.6
2	Square	230.2

## Data Availability

The original contributions presented in this study are included in the article. Further inquiries can be directed to the authors.

## Abbreviations

The following abbreviations are used in this manuscript:

EM	Energetic material
NERA	Non-explosive reactive armour
PBX	Plastic-bonded explosive
PETG	Poly(ethylene terephthalate) doped with glycol
RDX	1,3,5,-Trinitro-1,3,5-triazinane
VoD	Velocity of detonation
